# The burden of headache disorders in the adult population of Morocco: estimates, and a health-care needs assessment, from a cross-sectional population-based door-to-door survey

**DOI:** 10.1186/s10194-024-01942-9

**Published:** 2024-12-30

**Authors:** Najib Kissani, Latifa Adarmouch, Aboubacar Sidik Sidibe, Abderrahmane Garmane, Rachid Founoun, Mohamed Chraa, Andreas Husøy, Timothy J. Steiner

**Affiliations:** 1 Laboratory of Clinical and Experimental Neuroscience, Faculty of Medicine, Cadi Ayyad University, Marrakech, Morocco; 2 Department of Neurology, Mohammed VI University Hospital, Marrakech, Morocco; 3 Community Medicine and Public Health Department, Bioscience and Health Research Laboratory, Faculty of Medicine, Cadi Ayyad University, Marrakech, Morocco; 4 Marrakech Medical School, Marrakech, Morocco; 5 Department of Neuromedicine and Movement Science, Norwegian University of Science and Technology, Edvard Griegs gate, Trondheim, Norway; 6 Department of Neurology, University of Copenhagen, Copenhagen, Denmark; 7 Division of Brain Sciences, Imperial College London, London, UK

**Keywords:** Headache disorders, Migraine, Tension-type headache, Medication-overuse headache, Epidemiology, Burden of disease, Population-based survey, Morocco, Maghreb countries, Eastern Mediterranean Region, Global Campaign against Headache

## Abstract

**Background:**

We have previously shown headache disorders to be prevalent in in the adult general population of Morocco, especially migraine (30.8%) and headache on ≥ 15 days/month (H15+; 10.5%). This study, collecting data from the same population-based sample, is the first to estimate headache-attributed burden not only in Morocco but, more widely, in the Maghreb countries of North Africa.

**Methods:**

We used the standard methodology and questionnaire developed by the Global Campaign against Headache. Cluster-based random sampling generated a sample (*N* = 2,575) representative of the general population aged 18–65 years. Interviews conducted face-to-face enquired into symptom burden (headache frequency, usual duration and usual intensity), and impaired participation in paid work, household work and social or leisure activities during the preceding 3 months. Further enquiry was into headache yesterday (HY). We calculated population-level estimates by factoring in prevalence. Needs assessment estimated the population proportion in need of headache-related health care based on likelihood of benefit.

**Results:**

Participants with headache of any type spent, on average, 12.5% of their time with headache of intensity rated 2.3 on a scale of 1–3. According to age- and gender-corrected estimates, 7.2–8.4% of all time in the population (calculated by two methods) was spent with headache, H15 + accounting for well over half of this. Impaired participation measured as lost time due to headache averaged 0.5 days from paid work, 1.6 days from household work and 0.3 days from social or leisure activities during the preceding 3 months. Of those with HY (17.8% of the sample), 24.1% of males and 50.9% of females could do nothing or less than half of their planned activity yesterday. At population level this diluted to 7.0% of all activity lost to headache. At least 30% of the population were estimated to need headache-related health care.

**Conclusion:**

Headache disorders cause much ill health in the adult population of Morocco. While this will be of obvious concern to health policy in Morocco, the call for provision of health care for almost one third of this population is challenging. On the other hand, economic policy should recognise the lost-productivity costs of inadequately treated headache, especially migraine.

## Background

The series of population-based studies of headache-attributed burden conducted by the Global Campaign against Headache [1–12], using standardized methodology [13, 14], has so far included Pakistan [11, 15] and Saudi Arabia [16] from the Eastern Mediterranean Region (EMR). This study, using the same methodology, adds Morocco, one of the Maghreb countries of North Africa (the western part of the Arab world also including Algeria, Libya, Mauritania and Tunisia). While the Maghreb is part of EMR, it is geographically distant and ethnically and culturally very different from Pakistan and Saudi Arabia.

In an earlier publication, we reported 1-year prevalences in Morocco of migraine and tension-type headache (TTH), and of the disorders characterized by headache occurring on ≥ 15 days/month (H15+), including probable medication-overuse headache (pMOH) [17]. Both migraine (30.8%) and H15+ (10.5%) were found to be at the upper ends of the reported global ranges for these disorders, while the latter estimate was corroborated by the high proportion (17.8%) reporting headache on the day preceding the survey (“headache yesterday” [HY]) [17].

The coupling of measures of attributed burden with prevalence estimates is of essential importance to informed health policy. In addition to doing this, we undertook a basic health-care needs assessment, counting those in the population of Morocco aged 18–65 years who were likely to benefit from headache-related health care.

## Methods

We used data collected from the same participants and at the same time as the prevalence study [17]. The methods, previously described in detail [17], are summarised here.

### Ethics and approvals

The protocol and questionnaire were approved by the Comité d’Ethique pour la Recherche Biomédicale of Centre Hospitalier Universitaire Ibn Rochd Casablanca, Morocco. The study was conducted in accordance with the Declaration of Helsinki [18], and with Moroccan regulation concerning the exercise of medicine. All participants gave verbal consent to inclusion.

Administrative authorizations were required for house-to-house surveys, and were obtained from the regional administrative authorities of Agadir, Marrakech and Tétouan [17].

Data were gathered anonymously, and managed in accordance with data protection legislation [17].

### Study design and procedures

The study was a cross-sectional survey employing standardized methodology [13, 14], conducted between February and September 2019, which included the month of Ramadan (5 May to 4 June). We used four-level cluster sampling (region, district, household, individual), making unannounced visits to households in three geographical regions: Tétouan, Marrakech and Agadir. In a fourth (Fès), no response was received to our requests for administrative authorizations. This precluded unannounced household visits in this region. Interviews with 899 participants in Fès were conducted by other means (random encounter), which were in violation of the protocol [17]; therefore, no data from Fès are presented here.

Medical graduates or senior medical students, trained for the purpose and led by a study coordinator, interviewed one adult (18–65 years) randomly selected from among the occupants of each household (defined as a group of individuals living together and sharing a kitchen). Interviews followed the structured Headache-Attributed Restriction, Disability, Social Handicap and Impaired Participation (HARDSHIP) questionnaire [13]. After demographic and headache diagnostic enquiry (the latter based on ICHD-3), questions addressed symptom burden (headache frequency and usual intensity and duration) and impact of headache on participation in paid work, household work (meaning the necessary chores of daily life) and social or leisure activities (using the Headache-Attributed Lost Time [HALT] index [19], a module within HARDSHIP).

Participants reporting headaches of more than one type were asked to focus, during these enquiries, on the most bothersome type.

Headache diagnoses were imported from our previous study [17]. They had been algorithmically derived from the responses to HARDSHIP [13], with H15 + first identified (and classified as pMOH when associated with acute medication usage on ≥ 15 days/month, or as other H15+), then definite migraine, definite TTH, probable migraine or probable TTH in that hierarchical order [17]. Definite and probable diagnoses for migraine and for TTH were combined for burden analyses [14]. The few participants with unclassifiable headache (0.5%) were not included in the burden analyses.

In participants reporting HY, we recorded duration and intensity of HY, and impaired participation yesterday as “everything achieved”, “more than half achieved”, “less than half achieved” or “nothing achieved”.

### Statistics and analysis

Continuous data were collected for headache frequency (in days/month) and usual headache duration (in hours) and summarized for each headache type as means ± standard error of the mean (SEM) and medians. Data on headache intensity, reported categorically as “not bad”, “quite bad” or “very bad”, were converted into a numerical scale 1–3 and also treated as continuous. Average time spent with headache was calculated for each individual by multiplying attack frequency and duration (capped at 24 h), and expressed as a proportion of time in ictal state (pTIS) by dividing by 30*24. Lost health attributed to migraine and TTH was calculated as pTIS*DW, where DW was the disability weight from the Global Burden of Disease study [20] for the ictal state of each disorder, and expressed as a percentage.

Data on impaired participation in each activity domain were expressed as days lost per 3 months. For both paid and household work, in accordance with accepted methodology [19], days in which productivity was reduced by more than half (less than half achieved of what had been expected) were counted as whole days lost, and counterbalanced by ignoring days in which productivity was reduced by less than half. Estimates of overall impaired participation during HY, not distinguishing between activity domains, similarly summed counts of “nothing achieved” and “less than half achieved” yesterday and ignored “more than half achieved”. Where data were missing, we applied a value of zero (no days lost) in that domain (this is explained in Discussion).

We estimated sample-level pTIS as the product of mean individual-level pTIS and 1-year prevalence for each headache type. We corrected these estimates for age and gender to derive population-level estimates. We performed similar calculations for impaired participation based on HALT. In addition, we estimated population-level pTIS and overall impaired participation from HY data by factoring in 1-day prevalence, again correcting for age and gender.

Health-care needs assessment assumed that all those with attributed burden above threshold levels would be likely to benefit from care. We counted the following, avoiding double counting: all those with H15+ (pMOH or other); all those with migraine and a reported headache frequency of ≥ 3 days/month; all those with migraine or TTH reporting pTIS > 3.3% *and* usual intensity ≥ 2 (moderate or severe); all those with migraine or TTH reporting impaired participation in either paid or household work of > 3 days in the preceding 3 months. All estimates were corrected for age and gender to permit inferences at population level.

Comparisons of continuous variables were made using ANOVA tests, and of categorical variables using chi-squared tests. We set significance at *p* < 0.05.

Statistical analyses were performed using SPSS version 28 (SPSS, INC, Chicago, IL).

## Results

There were 2,575 participants, with a preponderance of females (1,535 [59.6%]) compared to the national population (49.7%; *p* < 0.001) [17]. In a second protocol violation, interviewers failed to record refusals, but retrospectively estimated the non-participating proportion as “up to 10%” [17]. Nevertheless, age and habitation distributions in the sample were reasonably matched to those of the national population. The age- and gender-adjusted 1-year prevalence estimates, already published but repeated here because they are used in our analyses, were 74.1% for all headache, 30.8% for migraine, 32.1% for TTH, 5.9% for pMOH and 4.6% for other H15+ [17].

**Table 1:** Symptom burden and lost health at individual level by headache type and gender

Headache type	Overall	Male	Female	Male vs. female
	Mean±SEM, median			
**Frequency** (days/month)				
Any headache	5.5±0.2, 2.0	4.2±0.2, 2.0	6.3±0.2, 3.0	F(1, 1936) = 37.7, *p* < 0.001
pMOH	22.0±0.5, 20.0	20.5±1.0, 20.0	22.4±0.6, 20.0	F(1, 154) = 2.4, *p* = 0.12
Other H15+	20.5±0.6, 18.0	21.4±1.1, 20.0	20.2±0.7, 18.0	F(1, 131) = 0.7, *p* = 0.40
Migraine	2.9±0.1, 2.0	2.5±0.1, 2.0	3.1±0.1, 2.0	F(1, 806) = 8.0, *p* = 0.005
TTH	2.9±0.1, 2.0	2.5±0.1, 2.0	2.8±0.1, 2.0	F(1, 824) = 2.8, *p* = 0.09
**Duration** (hours)				
Any headache	18.8±0.7, 12.0	13.9±0.7, 6.0	21.5±0.9, 24.0	F(1, 1774) = 30.9, *p* < 0.001
pMOH	20.5±1.6, 24.0	21.7±5.5, 24.0	20.2±1.5, 24.0	F(1, 153) = 0.1, *p* = 0.71
Other H15+	27.6±5.4, 24.0	13.4±2.0, 20.0	31.9±6.9, 24.0	F(1, 124) = 02.1, *p* = 0.15
Migraine	23.0±1.0, 24.0	16.4±1.0, 12.0	26.2±1.3, 24.0	F(1, 739) = 23.7, *p* < 0.001
TTH	12.7±0.7, 4.0	11.5±0.9, 3.5	13.7±1.1, 4.0	F(1, 741) = 2.4, *p* = 0.12
**Intensity** (mild, moderate, severe, equated to 1, 2, 3)				
Any headache	238-965-717 (mean = 2.3)	124-388-186 (mean = 2.1)	114-577-531 (mean = 2.3)	*X*^*2*^(2, *N* = 1919) = 65.9, *p* < 0.001
pMOH	5-41-108 (mean = 2.7)	1-13-17 (mean = 2.5)	4-28-91 (mean = 2.7)	*X*^*2*^(2, *N* = 154) = 4.7, *p* = 0.10
Other H15+	8-47-77 (mean = 2.5)	3-15-13 (mean = 2.3)	5-32-64 (mean = 2.6)	*X*^*2*^(2, *N* = 131) = 4.6, *p* = 0.10
Migraine	31-353-420 (mean = 2.5)	13-133-114 (mean = 2.4)	18-220-306 (mean = 2.5)	*X*^*2*^(2, *N* = 804) = 11.1, *p* = 0.004
TTH	192-522-106 (mean = 1.9)	106-226-40 (mean = 1.8)	85-296-66 (mean = 2.0)	*X*^*2*^(2, *N* = 819) = 11.3, *p* = 0.004
**Proportion of time in ictal state** (%)				
Any headache	12.5±0.6, 3.3	7.3±0.6, 1.6	15.3±0.8, 3.3	F(1, 1771) = 49.4, *p* < 0.001
pMOH	56.3±2.8, 60.0	44.1±5.6, 46.7	59.3±3.1, 63.7	F(1, 153) = 4.9, *p* = 0.03
Other H15+	50.6±3.0, 50.0	38.4±6.3, 50.0	54.3±3.3, 50.0	F(1, 124) = 5.2, *p* = 0.02
Migraine	6.0±0.3, 3.3	4.3±0.4, 2.2	6.8±0.4, 3.2	F(1, 738) = 18.3, *p* < 0.001
TTH	3.4±0.2, 1.1	3.4±0.3, 1.1	3.5±0.3, 1.4	F(1, 739) = 0.1, *p* = 0.71
**Lost health** (%)				
Migraine	2.6±0.1, 1.4	1.9±0.2, 1.0	3.0±0.2, 1.4	F(1, 738) = 18.3, *p* < 0.001
TTH	0.1±0.0, 0.0	0.1±0.0, 0.0	0.1±0.0, 0.1	F(1, 739) = 0.1, *p* = 0.71

pMOH: probable medication-overuse headache; H15+: headache on ≥ 15 days/month; TTH: tension-type headache

**Table 2:** Impaired participation (days/3 months) in paid and household work and social or leisure activities at individual level by headache type and gender

Headache type	Overall	Male	Female	Male vs. female
	Mean±SEM, median			
**Paid work (HALT questions 1 + 2)**				
Any headache	0.5±0.1, 0.0	0.7±0.1, 0.0	0.4±0.1, 0.0	F(1, 1936) = 15.9, *p* < 0.001
pMOH	1.4±0.4, 0.0	2.4±1.1, 0.0	1.1±0.4, 0.0	F(1, 154) = 1.8, *p* = 0.18
Other H15+	0.9±0.3, 0.0	2.8±1.0, 0.0	0.2±0.2, 0.0	F(1, 131) = 15.9, *p* < 0.001
Migraine	0.5±0.1, 0.0	0.8±0.2, 0.0	0.3±0.1, 0.0	F(1, 806) = 013.6, *p* < 0.001
TTH	0.3±0.1, 0.0	0.5±0.1, 0.0	0.2±0.1, 0.0	F(1, 824) = 5.3, *p* = 0.02
	F(3, 1920) = 10.6, *p* < 0.001			
**Household work (HALT questions 3 + 4)**				
Any headache	1.6±0.1, 0.0	0.4±0.1, 0.0	2.4±0.2, 0.0	F(1, 1936) = 90.2, *p* < 0.001
pMOH	6.5±0.8, 0.0	1.3±0.8, 0.0	7.8±0.9, 2.0	F(1, 154) = 12.5, *p* < 0.001
Other H15+	4.8±0.6, 2.0	1.3±1.0, 0.0	5.9±0.7, 3.0	F(1, 131) = 10.0, *p* = 0.002
Migraine	1.5±0.1, 0.0	0.5±0.2, 0.0	1.9±0.2, 0.0	F(1, 806) = 35.3, *p* < 0.001
TTH	0.4±0.1, 0.0	0.2±0.1, 0.0	0.6±0.1, 0.0	F(1, 824) = 13.0, *p* < 0.001
	F(3, 1920) = 118.5, *p* < 0.001			
**Social or leisure activities (HALT question 5)**				
Any headache	0.3±0.0, 0.0	0.2±0.0, 0.0	0.4±0.0, 0.0	F(1, 1936) = 8.8, *p* = 0.003
pMOH	1.3±0.3, 0.0	1.0±0.6, 0.0	1.3±0.4, 0.0	F(1, 154) = 0.2, *p* = 0.65
Other H15+	1.0±0.2, 0.0	1.1±0.6, 0.0	1.0±0.3, 0.0	F(1, 131) = 0.4, *p* = 0.84
Migraine	0.3±0.0, 0.0	0.2±0.0, 0.0	0.4±0.0, 0.0	F(1, 806) = 5.5, *p* = 0.02
TTH	0.1±0.0, 0.0	0.1±0.0, 0.0	0.1±0.0, 0.0	F(1, 824) = 1.4, *p* = 0.23
	F(3, 1920) = 37.5, *p* < 0.001			

HALT: headache-attributed lost time; pMOH: probable medication-overuse headache; H15+: headache on ≥ 15 days/month; TTH: tension-type headache

## Burden at individual level

### Symptom burden

Symptom burden and lost health are summarized in Table 1. Frequency of any headache was 5.5 days/month. Mean duration was 18.8 h and intensity 2.3 on the scale of 1–3 (moderate-to-severe).

Migraine and TTH had similar frequencies (2.9 days/month), but migraine was of much longer duration (23.0 vs. 12.7 h); accordingly, pTIS was higher for migraine (6.0% vs. 3.4%). Migraine was also more severe (2.5 vs. 1.9). Lost health (pTIS*DW) attributed to migraine was 2.6% (equivalent to a permanent diminution of 2.6% from a state of perfect health); lost health attributed to TTH was 0.1% (at the lower limit of estimation) (Table 1).

Frequencies of pMOH and other H15 + were, as expected, much higher (22.0 and 20.5 days/month). With reported durations also high (20.5 and 27.6 h), pTIS estimates exceeded 50% for both (i.e., more than half of all time was spent with headache). Both pMOH and other H15 + were rated on a par with migraine for intensity (Table 1).

**Fig. 1:**
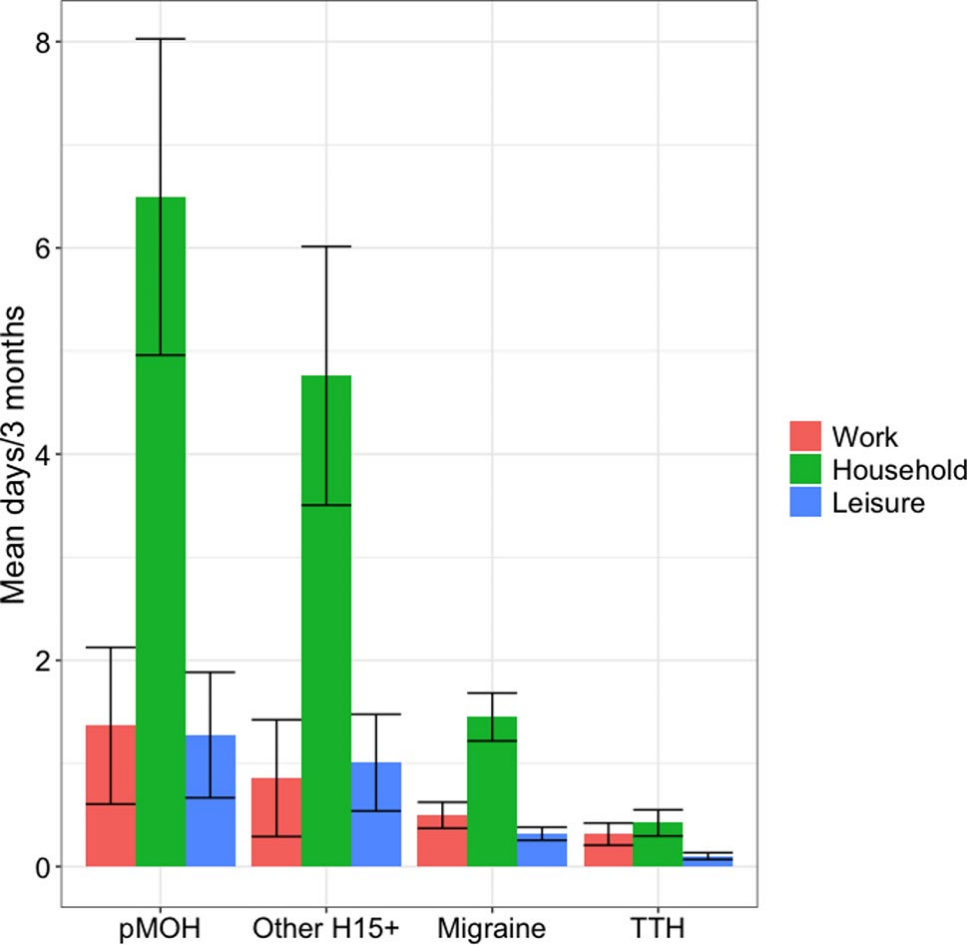
Lost days from paid work (red), household work (green) and social or leisure activities (blue) by headache type. pMOH: probable medication-overuse headache; H15+: headache on ≥ 15 days/month; TTH: tension-type headache

For all headache (*p* < 0.001) and for migraine (*p* < 0.005), females reported greater symptom burden than males in all measures. For all headache, pTIS in females was more than double that in males (15.3% vs. 7.3%) (Table 1).

### Impaired participation

Headache-attributed impaired participation by headache type and gender is summarized in Table 2 and Fig. 1.

The average lost times attributed to headache among those with any headache were 0.5 days in 3 months from paid work, 1.6 days from household work and 0.3 days from social or leisure activities. Losses attributed to migraine among those with migraine mirrored these, while those attributed to TTH among those with TTH were much lower (Table 2). Considerably higher losses were attributed to pMOH (1.4 days/3 months lost from paid work, 6.5 from household work and 1.3 from social or leisure activities), and to other H15+ (0.9, 4.8 and 1.0 days/3 months) (Table 2). Headache type was therefore significantly associated with impaired participation in all three domains (*p* < 0.001) (Table 2; Fig. 1). Males lost about twice as much time from paid as from household work, regardless of headache type, while females lost several-fold more time from household than from paid work (Table 2). Accordingly, males lost more time than females from paid work, significantly for all headache types but pMOH, while females lost more time than males from household work regardless of headache type. Females with migraine also lost more time than males from social or leisure activities. But, it should be noted, medians of zero indicated that more than half of males reported no impairment of participation in any domain, as did more than half of females with migraine or TTH.

**Table 3:** Symptom burden and impaired participation associated with headache yesterday

	Overall	Male	Female	Male vs. female
**Duration** (hours) mean±SEM, median	10.8±0.5, 6.0	7.8±0.8, 4.0	11.7±0.5, 8.0	F(1, 451) = 13.8, *p* < 0.001
**Intensity**				
mild (n) moderate (n) severe (n) mean*	117 268 80 (mean = 1.9)	32 67 15 (mean = 1.9)	85 201 65 (mean = 1.9)	*X*^*2*^(2, *N* = 463) = 2.0, *p* = 0.37
**What done**				
everything (n) more than half (n) less than half (n) nothing (n)	171 90 61 146	66 22 7 21	105 68 54 125	*X*^*2*^(3, 466) = 31.9, *p* < 0.001

* Equating to 1, 2, 3, and treating as though continuous data

**Table 4:** Proportion of time in ictal state and impaired participation at population level, by headache type and by timeframe of enquiry (adjusted for age and gender)

Headache type	Estimated pTIS (%)		Estimated impaired participation			
	According to 1-year prevalence and reported average frequency and usual duration	According to prevalence and duration of headache yesterday	According to HALT data (lost days/3 months)			According to headache yesterday
			Lost productivity		Social or leisure activities	Total impaired participation (%)
			Paid work	Household work		
Any headache	8.4	7.2	0.4	1.1	0.2	7.0
Migraine	1.7		0.2	0.4	0.1	
Tension-type headache	1.1		0.1	0.1	0.0	
Probable medication-overuse headache	3.0		0.1	0.3	0.1	
Other headache on ≥ 15 days/month	2.3		0.1	0.2	0.0	

pTIS: proportion of time in ictal state

### Headache yesterday

Symptom burden and overall impaired participation associated with HY are summarized in Table 3. As previously published, HY was reported by 17.8% of the sample [17].

On average, HY lasted 10.8 h (longer in females [11.7 h] than males [7.8 h; *p* < 0.001]) and was rated moderate. Impaired participation with HY (nothing or less than half done yesterday) was reported by 44.2%, with a two-fold difference between males (24.1%) and females (50.9%; *p* < 0.001) (Table 3).

### Population-level burden

Age and gender-adjusted headache-attributed pTIS and impaired participation at population level are summarized in Table 4.

With regard to symptom burden, pTIS at population-level calculated from 1-year prevalence, headache frequency and usual headache duration was 8.4% for all headache, 1.7% for migraine, 1.1% for TTH, 3.0% for pMOH and 2.3% for other H15+. Population-level pTIS for all headache estimated from HY was similar (7.2%).

**Table 5:** Health-care needs assessment

Criterion fulfilled		Proportion of sample		Estimated proportion of adult population*
		**n**	%	% [95% CI]
1	Headache on ≥ 15 days/month	289	11.2	10.5 [9.4–11.8]
2	Migraine on ≥ 3 days/month	353	13.7	13.2 [11.9–14.6]
3	Migraine and pTIS > 3.3% and moderate-severe intensity	294^1^	11.4	10.6 [9.5–11.9]
4	Migraine and lost work and/or household days/3 months ≥ 3	201^2^	7.8	7.0 [6.1–8.1]
5	TTH and pTIS > 3.3% and moderate-severe intensity	153	5.9	5.8 [4.9–6.8]
6	TTH and lost work and/or household days/3 months ≥ 3	67^3^	2.6	2.5 [1.9–3.2]
One or more of criteria 1–6		1,005	39.0	36.8 [34.9–38.7]

*Age- and gender-corrected; ^1^of whom 225 also fulfilled criterion 2; ^2^of whom 92 also fulfilled criterion 2, 77 also fulfilled criterion 3 and 59 also fulfilled criteria 2 and 3; ^3^of whom 17 also fulfilled criterion 5

With regard to impaired participation, average lost days at population level (i.e., per person with or without headache) for all headache during the preceding 3 months were 0.4 from paid work, 1.1 from household work and 0.2 from social or leisure activities (Table 4). Migraine accounted for most lost time from work (paid and household), followed by pMOH (Table 4). Population-level impaired participation based on HY data, for all headache, was 7.0%.

### Health-care needs assessment

Of the 2,575 participants, a total of 1,005 (39.0%) had a headache disorder that met our criteria for likelihood of benefit from health care. Half of these (513) had migraine, 289 had H15 + and 203 had TTH (Table 5). Adjusted for age and gender, need for headache-related health care in Morocco was estimated to exist in 36.8% of the population aged 18–65 years (18.9% for migraine, 10.2% for H15 + and 7.7% for TTH). There is discussion of this proportion below.

## Discussion

This is the first study to estimate the burden attributable to headache disorders in the adult general population of Morocco, the first in the Maghreb countries of North Africa, and the third of its type in EMR (after Pakistan [15] and Saudi Arabia [16]). It used the standard methodology and questionnaire developed by the Global Campaign against Headache [13, 14]. It found the symptom burden to be high: people with headache of any kind spent, on average, 12.5% of their time with headache of intensity rated 2.3 on a scale of 1–3. Females had significantly higher symptom burden than males. Average lost time attributed to headache was 0.5 days in 3 months from paid work, 1.6 days from household work and 0.3 days from social or leisure activities. Losses from paid work were considerably higher among males than females, a reflection of the low participation of women in the labour force in Morocco (among the lowest globally [21]). Losses from household work, on the other hand, were – as expected – significantly higher among females than males. A quarter of males (24.1%) and half of females (50.8%) with HY (17.8% of the sample) reported nothing or less than half done yesterday. At population level this diluted to an estimated 7.0% of all activity lost to headache.

These findings add to those on prevalence from our earlier study [17], informing health policy. In that study, we commented at length on the very high prevalence of migraine (30.8%) in comparison with the estimated global prevalence of 14–15% [22]. Inclusion of probable migraine was certainly a factor, and interest bias (mentioned again below) might have been [17]. Burden estimates at population level are, of course, to a large extent driven by prevalence estimates.

The high symptom burden at population level (8.6% of all time in the adult population spent with headache) is indicative of a high level of health-care need, but also offers the prospect of substantial improvement in population health through provision of health care to meet this need (effective treatments exist, and are cost-effective [23–25]). Our criteria for likelihood of benefit identified more than one third (36.8%) of the adult population of Morocco in need, a very similar proportion to the 35.8% we found in the Kingdom of Saudi Arabia [16] – despite the differences in culture, ethnicity and wealth. Half of those in Morocco (18.9%) were because of troublesome migraine (frequent and/or associated with substantially impaired participation) and more than one quarter (10.2%) were because of H15+. But 7.7% were because of TTH, either with pTIS > 3.3% and moderate or severe headache or with impaired participation in either paid or household work of > 3 lost days in 3 months. This last group need treatment, but, arguably, this need can largely be met by appropriate use of over-the-counter medications: that is, by health education and advice from community pharmacists rather than medical care. With these in place, health-care need would be reduced to about 30% of the adult population.

Our findings also inform economic policy in Morocco. Impaired participation estimates suggest that the health benefits of effective health care for those with disabling headache would be accompanied by the economic benefits of higher production. Means and medians were very far apart for lost-productivity findings (lost days from paid and household work), indicating a high level of skewedness, which suggests that a minority – who might be a priority target for health care –contribute disproportionately to population-level productivity losses. As expected, given the differences in symptom burden, higher productivity losses at individual level were attributable to migraine and H15 + than to TTH, and higher losses from paid work were attributable to H15 + than to migraine (Fig. 1). Nonetheless, while pMOH and other H15 + were associated with far higher pTIS than migraine at both individual and population levels, and were of similar intensity to migraine, it was migraine that was associated with the greatest productivity losses at population level. In other words, from an economic perspective, it is migraine that appears to be the priority target for health care. Migraine alone, it should be noted, was associated in those affected with the equivalent of a permanent diminution in health of 2.6%.

However, structured headache services – the suggested health-care solution to the burden of headache [23] – provide care not for specific headache types but for the full range of primary headache disorders, and MOH, and are cost-effective when priority is determined clinically rather than driven by expected economic benefit [24, 25].

This said, in many economies, structured headache services are potentially cost saving through recovery of lost productivity [25]. In Morocco, for migraine, pMOH and other H15+, productivity losses were greater from household work than from paid work, although significantly only for migraine (Fig. 1). This is unsurprising, since losses from household work were driven by females, among whom headache disorders were more prevalent. It is also the case that household work can, generally, be more easily deferred than paid work. However, household work means the necessary everyday chores of living, and its economic value should not be underestimated.

Recall error is a factor to be considered when disease-attributed burden is estimated from participants’ recall of symptoms and their effects on activities over past months. Accordingly, we made separate estimates based on yesterday (1-day recall) [13, 14]. With regard to population-level pTIS, the two estimates for all headache were similar: 7.2% based on HY and 8.4% based on long recall. This suggests our finding is reliable that the proportion of all time spent with headache in Morocco (among the population aged 18–65 years) is in this range 7.2–8.4%.

In contrast, headache-attributed impaired participation reported in association with HY (7.0%) was apparently much higher than impaired participation recalled over the preceding 3 months. But comparisons here were indirect. Impaired participation yesterday with HY was reported as how much, of planned activity, actually was not done, expressed as a proportion (%) without differentiating between different domains of activity. HALT-based enquiry, over the preceding 3 months, counted days lost in each domain separately, but it did not provide denominators for reliable calculation of proportionate losses. If it were assumed that, in 3 months, there were 65 days of paid work that could be lost, 90 days of household work and 90 days of social or leisure activities (allowing that, in any single day, activities might have been planned in all three domains), and if losses in the three domains were summed, the proportion would be 2.1% {([0.4/65]+[1.1/90]+[0.2/90])*100} – much lower than the 7.0% estimated from HY data. But such assumptions would be untenable: the denominators are not knowable. For those engaged in paid work, denominators might be larger or smaller than 65 days, while denominators for household work and social or leisure activities might be (very much) smaller than 90 days. Further, in the HALT-based enquiry, participants were likely not to respond when an activity domain appeared irrelevant (for example, paid work for most females). We applied values of zero whenever data were missing (see Methods), so that individual-level averages were for all participants.

Therefore, the two methods of estimating impaired participation, rather than being discrepant, yield different information: absolute and relative. Lost productivity is better estimated from HALT data, but only in absolute terms: as average lost days per person (with or without headache). Overall impaired participation among the population (again including those with or without headache) is better estimated, as a proportion, from HY data.

As noted, data were collected during February to September 2019, which period included Ramadan (5 May to 4 June), in a country that is very predominantly Muslim. Dates of interview of each individual participant were not recorded, but about 12% of the data (one month out of eight) would have been collected during Ramadan. There is evidence that fasting is a cause of TTH-like headache [26], and, more specifically (although clinic- rather than population-based [27, 28]), that fasting during Ramadan has an aggravating effect on migraine, albeit, perhaps, only during the first 10 days [28]. Heightened awareness of headache during the fasting of Ramadan is also possible. Any such effects of Ramadan would have been captured in our study, with slight overemphasis (one month out of eight, rather than twelve). But these effects appear to be both small and uncertain, so that significant overestimation of overall burden is very unlikely.

The strengths of this study are that it was population-based, with an adequate sample size [14] (*N* = 2,575), and conducted in accordance with standardized methodology [13, 14]. While the sample had a high female-to-male ratio, statistical corrections for this were possible. Burden and productivity measures were assessed over short (1 day) as well as long time periods (weeks to 3 months) in order to rectify recall error.

Some limitations, previously noted [17], must also be considered. The failure of the interviewers to record refusals was a protocol violation. Their later recall of “up to 10%” cannot be relied on. Interest bias becomes likely when the non-participating proportion is large (> 10%), but, as noted previously [17], the observed 1-year prevalence of all headache (75.3%) was not unusually high. The necessary exclusion of the Fès sample because of the inappropriate method of sampling did not appear to have great impact on representativeness since the previously reported prevalence findings in this sample, with age- and gender-adjustment, were not greatly different from those of the main sample [17]. In cross-sectional studies with enquiry at a single encounter, further diagnosis of H15 + is limited to recognising its association with acute medication overuse (pMOH), or not (other H15+) [10, 11]. The diagnostic algorithm allowed only one diagnosis per participant, with migraine more likely to be diagnosed when both migraine and TTH were present. TTH-attributed burden might, therefore, have been somewhat underestimated. Resources were not available to validate the diagnostic question set within this study, but it had been validated in six earlier studies [15, 29–33] and used previously in 19 languages [13] including both French [34] and Arabic [10].

## Conclusion

Headache disorders in the adult general population of Morocco are associated with high levels of attributed symptom burden and ill health. They substantially impair participation in work and social activities, and are detrimental to productivity and, inevitably therefore, to individual and national economies. They create need for health care in at least 30% of this population. Health and economic policies in Morocco might note that investment in meeting this need, through national structured headache services [23], could expect return in the form of large health gains, while the cost would be offset by the productivity gains [24, 25].

## Data Availability

The original data are held on file at Marrakech Medical School, Marrakech, Morocco, and the analytical dataset at Norwegian University of Science and Technology, Trondheim, Norway. Once analysis and publications are completed, they will be freely available for non-commercial purposes to any person requesting access in accordance with the general policy of the Global Campaign against Headache.

## Abbreviations

ANOVA: Analysis of variance
CI: Confidence Interval
d/m: Days/month
DW: Disability weight
EMR: Eastern Mediterranean Region
HALT: Headache-attributed lost time (index)
HARDSHIP: Headache-Attributed Restriction, Disability, Social Handicap and Impaired Participation (questionnaire)
HY: Headache yesterday
ICHD: International Classification of Headache Disorders
HY: Headache yesterday
MOH: Medication-overuse headache
pMOH: Probable MOH
pTIS: Proportion of time in ictal state
SEM: Standard error of the mean
TTH: Tension-type headache
